# Similar Seed Composition Phenotypes Are Observed From CRISPR-Generated In-Frame and Knockout Alleles of a Soybean *KASI* Ortholog

**DOI:** 10.3389/fpls.2020.01005

**Published:** 2020-07-08

**Authors:** Kamaldeep S. Virdi, Madison Spencer, Adrian O. Stec, Yer Xiong, Ryan Merry, Gary J. Muehlbauer, Robert M. Stupar

**Affiliations:** Department of Agronomy & Plant Genetics, University of Minnesota, Saint Paul, MN, United States

**Keywords:** soybean, *KASI*, sucrose, oil, CRISPR, Cas9, seed, mutant

## Abstract

The β-ketoacyl-[acyl carrier protein] synthase 1 (*KASI*) gene has been shown in model plant systems to be critical for the conversion of sucrose to oil. A previous study characterized the morphological and seed composition phenotypes associated with a reciprocal chromosomal translocation that disrupted one of the *KASI* genes in soybean. The principle findings of this work included a wrinkled seed phenotype, an increase in seed sucrose, a decrease in seed oil, and a low frequency of transmission of the translocation. However, it remained unclear which, if any, of these phenotypes were directly caused by the loss of *KASI* gene function, as opposed to the chromosomal translocation or other associated factors. In this study, CRISPR/Cas9 mutagenesis was used to generate multiple knockout alleles for this gene, and also one in-frame allele. These soybean plants were evaluated for morphology, seed composition traits, and genetic transmission. Our results indicate that the CRISPR/Cas9 mutants exhibited the same phenotypes as the chromosomal translocation mutant, validating that the observed phenotypes are caused by the loss of gene function. Furthermore, the plants harboring homozygous in-frame mutations exhibited similar phenotypes compared to the plants harboring homozygous knockout mutations. This result indicates that the amino acids lost in the in-frame mutant are essential for proper gene function. In-frame edits for this gene may need to target less essential and/or evolutionarily conserved domains in order to generate novel seed composition phenotypes.

## Introduction

Seed composition traits are critical for soybean end uses. The protein fraction is important for food uses and livestock feed and the oil fraction is useful for food, fuel, and industrial applications. Furthermore, the carbohydrate fraction can impact the end use of the bean, particularly in the development of varieties for human consumption. Therefore, a better understanding of the genes that govern the seed composition components will be useful to increase the breeding efficiency of desirable traits for commodity and specialty markets.

Recent work from our group identified a fast neutron-induced chromosomal translocation that co-segregated with an increased seed sucrose and reduced oil phenotype (Dobbels et al., 2017). This locus was defined by a reciprocal translocation between chromosomes 8 and 13, which disrupted an internal exon of a β-ketoacyl-[acyl carrier protein] synthase 1 (*GmKASI*) ortholog (soybean gene model Glyma.08G084300). The seeds homozygous for the translocation also exhibited a wrinkled phenotype, consistent with previous observations for mutants of this gene in *Arabidopsis thaliana* (Wu and Xue, 2010). Furthermore, the translocated *KASI* allele was observed to transmit and segregate at a frequency far below Mendelian expectations. This work left three major questions unresolved: (1) Could the seed composition function of the soybean *KasI* gene be validated using CRISPR mutagenesis, and would the phenotype be different from the fast neutron line? (2) Given the extreme nature of the seed composition phenotype in the fast neutron line, would it be possible to generate an intermediate (i.e., less severe) phenotype by generating an in-frame mutant allele for the *KASI* ortholog? (3) Was the reduced transmission of the *KASI* mutant a consequence of the knocked-out *kasI* allele, or did it result from abberant meiosis of the translocated chromosomes?

This study addresses these three questions. We used CRISPR/Cas9 gene editing methods to generate both an in-frame and knockout alleles of the soybean *KASI* ortholog. We observed both the segregation patterns of these alleles, and monitored the seed composition phenotypes of the segregating families. All alleles generated in this study did not have any chromosomal abnormalities at the *KASI* site, but rather exhibited relatively small nucleotide deletions and insertions, as are typical of CRISPR/Cas9 edited sites. We found that these mutant lines also exhibited reduced transmission of the *kasI* alleles, for both in-frame and knockout alleles. Furthermore, we observed similar seed phenotype profiles for the in-frame and the knockout alleles. One of our goals of this study was to generate in-frame mutants with intermediate phenotypes and higher (i.e., normal) transmission levels. However, it appears that the location and amino acids deleted from the in-frame mutant were critical for normal *KASI* function, and thus mimicked the knockout allele phenotypes.

## Materials and Methods

### CRISPR/Cas9 Design and Assembly and Soybean Whole Plant Transformation

Whole protein sequences of soybean KASI and the nearest Arabidopsis ortholog were compared to identify evolutionarily conserved domains that could be targeted for mutagenesis. The protein sequences were obtained from the Phytozome and TAIR websites, respectively. Both proteins were aligned with T-Coffee (https://www.ebi.ac.uk/Tools/msa/tcoffee/) and the aligned fasta file was visualized with BoxShade (https://embnet.vital-it.ch/software/BOX_form.html) software.

**Figure 1:**
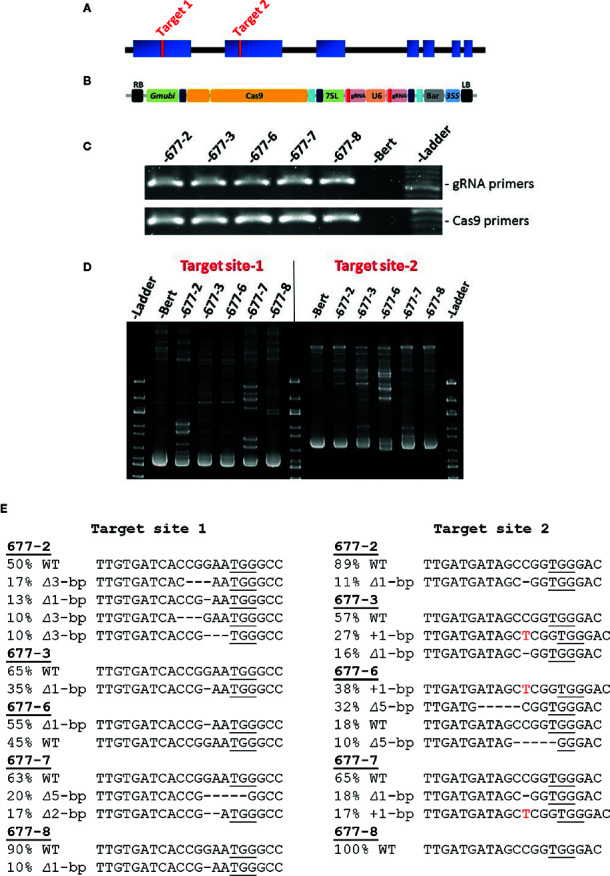
CRISPR/Cas9 targeting of soybean GmKASI and identification of mutations in T0M0 plants **(A)** Predicted gene model of *GmKASI* (Glyma.08g084300). Exons are depicted as blue rectangles. Red bars in exon 1 and exon 2 indicate the target sites for gene editing. **(B)** The T-DNA of the assembled CRISPR/Cas9 construct carrying two gRNAs, each targeting the respective sites shown in **(A)**. Cas9, gRNA for target-1, gRNA for target-2, and BASTA (glufosinate) selectable marker were driven by *Glycine* max ubiquitin (*Gmubi*), Arabidopsis ubiquitin (*U6*), *7sL*, and *35S* promoters, respectively (image adopted from Curtin et al 2018). **(C)** T-DNA presence in five independent T0M0 events (677-2, 677-3, 677-6, 677-7, 677-8) detected by PCR. Two sets of primer pairs were used, specific to the gRNA and Cas9 regions of the T-DNA. **(D)** Heteroduplex assays showed novel amplicon bands in the transformed plants compared to the ‘Bert’ wild-type control, indicating the presence of *GmKASI *edited alleles. **(E)** Sanger sequence analysis for all T_0_M_0_ events revealed various *GmKASI *edited alleles at both target sites. The genomic region spanning each target site was PCR amplified, Sanger sequenced, and analyzed with ICE software. Each T_0_M_0_ event showed a different proportion (indicated by %) of edited alleles (∆ indicates deletions; + indicates insertions). The underlined bases are the Protospacer Adjacent Motif (PAM) site at each target site.

Target sites for gRNAs were identified using the CRISPR-P 2.0 website (http://crispr.hzau.edu.cn/CRISPR2/; Liu et al., 2017). Oligos were synthesized from Integrated DNA Technologies (https://www.idtdna.com/pages). All CRISPR/Cas9 reagents were then assembled as described in Curtin et al. (2018). The final construct was transformed into the disarmed *Agrobacterium rhizogenes* strain 18r12 (Veena and Taylor, 2007). Whole plant soybean transformation was performed in the genetic background of the cultivar “Bert” (Orf and Kennedy, 1992), sub-line Bert-MN-01, using previously published methods (Paz et al., 2006; Liu et al., 2019). CRISPR/Cas9 reagents for whole plant transformation were assembled as described in Curtin et al. (2018). This included the Cas9-encoding sequence, two distinct gRNAs, and sequence encoding the glufosinate selectable marker. These components were driven by *Gmubi, U6, 7sL,* and *35S* promoters, respectively (**Figures 1A, B**).

### Polymerase Chain Reaction Targeted Amplicon for Heteroduplex Assays, CAPS Assays, and Sanger Sequencing for Detecting CRISPR/Cas9 Edits

The genomic regions spanning gRNA target sites were amplified by polymerase chain reaction (PCR) using the HotStarTaq Plus master mix (Qiagen, Hilden, Germany) according to the manufacturer's instructions. New mutations at the targeted sites were identified using either heteroduplex of Cleaved Amplified Polymorphic Sequences (CAPS) analyses. Heteroduplex assays were performed as previously reported (Zhu et al., 2014). For CAPS assays, targeted PCR amplicons were digested with BsaWI (New England Biolabs, Ipswich, MA) overnight at 60°C as per the manufacture's guideline. Digested products were run on agarose electrophoresis gels (1.3%). The presence of digestion-resistant PCR amplicons indicated CRISPR/Cas9 induced edits/mutations had occurred. Two approaches were used for Sanger sequencing. First, targeted PCR amplicons were directly sequenced and.abi files containing sequence information were then analyzed by Inference of CRISPR Edits (ICE) software (https://ice.synthego.com; Hsiau et al., 2018) to identify mutations. Alternatively, PCR amplicons were sub-cloned into the Topo TA cloning vector (Thermo Fisher Scientific, Waltham, MA) as per the manufacturer's instructions and individual positive clones were sequenced. Each sequence file (.abi) was visualized in ABI sequence scanner software. DNA sequence files were aligned with MultAlin software (http://multalin.toulouse.inra.fr/multalin/; Corpet, 1988).

### Whole Genome Sequencing and Bioinformatics

Selected plants were resequenced to confirm new mutations and the presence/absence of the transgene sequences. As both T-DNA and plasmid backbone sequences were integrated into the genome of the main T_0_ plant of interest (see Results section), henceforth these sequences will be distinguish using the terms “T-DNA” and “backbone,” rather than the term “transgene.”

DNA from young leaves at the second trifoliate stage was extracted with the DNeasy plant kit (Qiagen). Sequencing was performed at University of Minnesota Genomics Center to approximately 20x coverage per genotype. The T-DNA and plasmid backbone insertion sites were identified using the method of Michno et al. (2020) using the bash script TransGeneMap (https://github.com/MeeshCompBio/Soybean_Scripts) with the following modifications. Initial quality was assessed using Fastqc version 0.11.7 (Andrews, 2010). Trimmomatic version 0.33 (Bolger et al., 2014) was used for adapter removal, keeping a minimum read length of 40 bp and quality cutoff set to a phred score of 20. Read mapping to the soybean genome was conducted using bwa version 0.7.17 (Li and Durbin, 2010) following the same parameters as Michno et al. (2020). To locate the sites of integration in the genome, a FASTA file was generated using the entire sequence of the plasmid (including both the T-DNA and backbone) for read mapping, which allowed us to identify integration of the backbone as well as the T-DNA sequences. Orphan reads were mapped back to the soybean reference genome (Wm82.a2.v1) using bowtie2 version 2.3.4.1.

To discern the zygosity state of the vector backbone insertion, reads from sequenced plants were aligned to the soybean reference genome using the bash script Fastq2ReadmapGmaxV2 (https://github.com/MeeshCompBio/Soybean_Scripts) with the following modifications. Initial quality was again assessed using Fastqc version 0.11.7, adapter removal was done using cutadapt version 1.18 (Martin, 2011), and bwa version 0.7.17 was used for alignment. IGV version 2.3.97 (Robinson et al., 2011) was used to visually screen for the position and state of the vector backbone. Plants with reads aligning across the insertion site with reduced read depth at the site compared to the surrounding region were determined to be heterozygous. Plants with no reads aligning across the insertion site or mate pairs spanning the site (indicating a large insertion) were determined to be homozygous for the vector backbone insertion.

### Plant Materials, Growth, and Morphological Analysis

All plant materials were grown under similar growth conditions in a single greenhouse. The greenhouse temperature was maintained between 21–23°C. Natural light was supplemented with 600 watt high pressure sodium (HPS) lamps using a photoperiod day length of 14 h. All CRISPR/Cas9 edited and control plants were planted at the same time in a propagation mix growth medium (Sungro brand). Leaf samples for DNA extraction were harvested at the second trifoliate stage. Plants from different CRISPR/Cas9 families and control genotypes were randomly arranged. Plants were fertilized every 2 weeks using 400 ppm Jack's water-soluble 20-3-19 fertilizer until they reached the R7 stage. Plants were watered after every 2 d until the R7 stage and then reduced to once per week until the R8 stage. Individual plants were manually harvested and threshed to maintain genetic purity. Threshed seeds were kept in packets in the greenhouse for 1 week to promote drying. Transmissible mutations were identified from the progeny derived from three different T_0_ plants (see *Results*). A series of segregating mutant alleles was identified in the progeny of the T_0_ plant WPT677-3. Five of these families, each segregating for a distinct combination of alleles for the Glyma.08G084300 gene, were phenotyped in downstream seed composition analysis (see *Near Infrared Scan for seed Composition*), along with a control family that was homozygous for the wild-type allele (**Table 1**). Furthermore, seedling growth rates were measured for two homozygous mutant lines compared to homozygous wild-type siblings; ten seedlings were grown and measured from each genotype.

**Table 1:** Status of targeted mutations, T-DNA presence/absence, and plasmid backbone presence/absence in a sub-set of CRISPR/Cas9 confirmed to carry mutations in *GmKASI*.

Plant	Gen.	Glyma.08G084300	T-DNA			Plasmid backbone		
		Edits (site1.site2)	Status	Chr	Integration	Status	Chr	Integration
WPT677-3	T_0_M_0_	Various	Heteroyg.	5	5839464. 5839707	Heterozyg.	8	17781297. 17781301
WPT677-3-35	T_1_M_1_	+10/+107.WT/WT^a^	Absent	N/A	N/A	Heterozyg.	8	17781297. 17781301
WPT677-3-43	T_1_M_1_	WT/Δ1.WT/Δ1^b^	Absent	N/A	N/A	Heterozyg.	8	17781297. 17781301
WPT677-3-44	T_1_M_1_	Δ1/+1.WT/+1^a^	Absent	N/A	N/A	Heterozyg.	8	17781297. 17781301
WPT677-3-47	T_1_M_1_	WT/WT.WT/WT^c^	Absent	N/A	N/A	Heterozyg.	8	17781297. 17781301
WPT677-3-48	T_1_M_1_	WT/Δ6.WT/+1^b^	Absent	N/A	N/A	Homozyg.	8	17781297. 17781301
WPT677-3-22-04	T_2_M_2_	WT/Δ6.WT/+1^b^	Absent	N/A	N/A	Heterozyg.	8	17781297. 17781301
WPT677-3-22-10^e^	T_2_M_2_	WT/Δ6.WT/WT^d^	Absent	N/A	N/A	Untested		

a Biallelic knockout genotype.

b Heterozygous knockout genotype.

c Homozygous wild-type genotype.

d Heterozygous in-frame genotype.

e Genotype WPT677-3-22-10 was not subjected to WGS. The edited alleles were determined by Sanger sequencing and the absence of the T-DNA sequence was based on PCR analysis (see Results section).

Δ = deletion.

+ = insertion.

### Near Infrared Scan for Seed Composition

Two growouts were performed for seed composition analysis of the segregating families derived from WPT677-3 (**Table 1**). Within each of these two experiments, three to 17 biological replicates (the median number of plants among the mutant families was eight) were measured for each mutant family (detailed information on the number of plants in each family per experiment are provide in **Supplementary Tables S1** and **S2**). As these families were segregating, each plant was genotyped and grouped into the appropriate mutant class (homozygous mutant, heterozygous mutant, or homozygous wild-type) for seed composition analysis. Approximately 20 g of whole soybean seeds from individual plants were ground to a fine powder using a water cooled Foss KN195 Knifetec rotary grinder. The seeds were exposed to three consecutive pulses of grinding for 10 s each (30 s total) while rocking the grinder to ensure that all the seed material was ground. After completion, the material was removed from the grinder and immediately placed in a bag and immediately vacuum sealed until near infrared (NIR) scanning was performed. NIR scans and calculations of predicted values for each seed composition trait were computed as previously described (Dobbels et al., 2017).

### Statistical Analysis

All statistical analyses were conducted in R 3.5.2 (R Core Team, 2018, https://www.r-project.org/) and figures were produced with ggplot2_3.2.1 package (Wickham, 2016).

### Data Availability

Sequence data have been deposited into the NCBI Short Read Archive under the Bioproject identifier PRJNA640373.

## Results

### CRISPR/Cas9 Induced Targeted Mutagenesis of the Soybean *KASI* (*GmKASI*)

The predicted soybean gene model for Glyma.08G084300, henceforth referred to as *GmKASI*, indicates seven exons (https://soybase.org/; **Figure 1A**). To determine the guide RNA (gRNA) target sites for CRISPR/Cas9 gene editing, we first compared the protein sequences of *GmKASI* and its Arabidopsis ortholog. The genes are highly conserved, with 86.5% identity at the amino acid level (**Supplementary Figure S1**). Based on the conserved regions of the protein, two gRNA target sites were selected, one in exon 1 (target site-1) and second in exon 2 (target site-2; **Figure 1A**).

The resulting construct (**Figure 1B**) was transformed into soybean, resulting in five T_0_ plants: WPT677-2, WPT677-3, WPT677-6, WPT677-7, and WPT677-8. PCR assays with primers specific to gRNA and Cas9 regions of the T-DNA detected the presence of transgene sequences in all T_0_ plants (**Figure 1C**). A heteroduplex assay was conducted for both target sites to test for the presence of mutations. Heteroduplex assays involve melting and subsequent renaturation of the target site PCR amplicons prior to electrophoresis. If the homologous gene copies have different sequences due to new mutations, then the renatured DNA will have some imperfect double-stranded complexes, resulting in slower migration and thus novel bands observed on the gel. The heteroduplex assays for the T_0_ plants all showed novel amplicon bands, indicating new mutations occurred in the T_0_ plants (**Figure 1D**). Therefore, this generation was renamed as T_0_M_0_. Sanger sequencing of the PCR products from each target region confirmed various edited alleles of *GmKASI* in these plants (**Figure 1E**). To further confirm the mutations, sub-cloned target site-1 amplicons from plants WPT677-2, WPT677-3, and WPT677-6 were sequenced (**Supplementary Figure S2**). These assays confirmed editing at the site, though at a relatively low frequency. CAPS assays at target site-1 revealed digestion-resistant bands in all T_0_M_0_ plants, further confirming mutated alleles of *GmKASI* (**Supplementary Figure S3**).

### Generation of Stable and Heritable Knockout and In-Frame Mutant Alleles of *GmKASI* in Soybean

CAPS assays and Sanger sequencing were used to screen the inheritance of mutations in subsequent generations. CAPS assays on T_1_M_1_ families of WPT677-2 and WPT677-6 plants showed that the targeted mutations were successfully transmitted, as both families segregated for wild type and mutant alleles (**Supplementary Figure S4**). All T_1_M_1_ plants from both families also inherited T-DNA sequences, as detected by PCR. This finding indicates that multiple unlinked copies of T-DNA were likely integrated in the genome during transformation. However, the WPT677-7 and WPT677-8 families did not inherit the T-DNA nor mutations in the T_1_M_1_ generation (**Supplementary Figure S5**), suggesting the T-DNA did not stably integrate into the genome and all mutations observed in the T_0_M_0_ generation occurred in somatic cells.

Both mutations and T-DNA sequences were inherited in the T_1_M_1_ generation of plant WPT677-3 (**Supplementary Figure S6**; **Supplementary Table S3**). In this family, the mutations and T-DNA sequences appeared to segregate independently in this generation. A nearly 3:1 segregation ratio was observed for the T-DNA sequences (32 plants carrying T-DNA: 13 plants not carrying T-DNA). This finding suggested the T-DNA insertion in the T_0_M_0_ plant was likely a single copy event. The WPT677-3 lineage was subjected to further analyses in the T_1_M_1_ and T_2_M_2_ generations to identify individuals with heritable and stable targeted mutations in the absence of the CRISPR/Cas9 T-DNA. We selected two homozygous mutant plants (WPT677-3-35, WPT677-3-44), three heterozygous plants (WPT677-3-43, WPT677-3-48, WPT677-3-22-10), and one homozygous wild type plant (WPT677-3-47). A PCR based assay did not detect the presence of the T-DNA in any of these plants.

**Figure 2:**
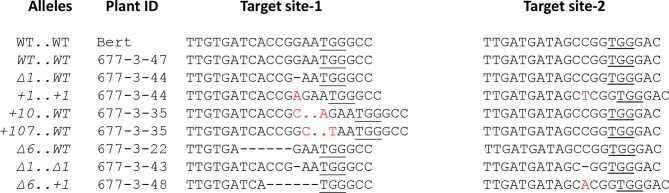
Isolation of multiple stable alleles of *GmKASI* inherited in T_1_M_1_ plants Eight independent *GmKASI* alleles with a combination of mutations at target site 1 and target site 2 were inherited in the T_1_M_1_ progeny of the 677-3 T_0_M_0_ plant. Each target site was amplified by Polymerase Chain Reaction and Sanger sequenced. Plant 677-3-47 carried homozygous wild-type allele. Plants 677-3-35 and 677-3-44 were biallelic, carrying two heterozygous mutant alleles, and are thus shown twice (∆ indicates deletions; + indicates insertions; red letters indicate inserted bases). The “..” in the plant 677-3-35 alleles indicates additional inserted bases not shown. 677-2-22, 677-3-43, and 677-3-48 each carried one mutant allele. The *GmKASI *alleles are depicted as *mutation attarget site 1* .. *mutation* at target site 2. The underlined bases are the Protospacer Adjacent Motif (PAM) site at each target site.

Detailed Sanger sequence analysis of plants WPT677-3-35, WPT677-3-44, WPT677-3-43, WPT677-3-48, and WPT677-3-22-10 revealed various edited alleles of *GmKASI* at both target sites (**Figure 2**). We performed Whole Genome Sequencing (WGS) of the T_0_M_0_ plant (WPT677-3), five T_1_M_1_ plants (WPT677-3-35, WPT677-3-43, WPT677-3-44, WPT677-3-47, WPT677-3-48), and one T_2_M_2_ plant (WPT677-3-22-04). At the time of the WGS experiment, the WPT677-3-22-10 plant was not available so we instead sequenced WPT 677-3-22-04, a sibling that did not carry the T-DNA sequences. No mutations were detected in WPT677-3-47, which served as the unedited control in these experiments. Notably, novel mutations at both target sites in the T_1_M_1_ generation were detected which were not present in the parental T_0_M_0_ plant (WPT677-3). One possible explanation is that heritable mutations continued to occur after the WPT677-3 leaves were sampled for the initial analysis. We detected two plants carrying biallelic mutations (WPT-677-3-35 and WPT677-3-44), two plants carrying heterozygous knock out alleles (677-3-43 and 677-3-48), and one plant carrying a heterozygous in-frame allele (WPT677-3-22-10) (**Table 1**). We detected edits at both target sites in WPT677-3-22-04, resulting in another knock out allele (**Table 1**); we did not follow this line for phenotypic analysis. All of the edited alleles were stably inherited to the T_2_M_2_ generation of WPT677-3-35, WPT677-3-44, WPT677-3-43, WPT677-3-44, WPT677-3-48, and the T_3_M_3_ generation of WPT677-3-22-10 (**Supplementary Tables S1** and **S2**).

Whole genome analysis confirmed that only the T_0_M_0_ plant contained the T-DNA sequences, which were integrated on chromosome 5 (**Table 1**). All of the selected progeny plants were confirmed to lack the T-DNA, indicating that this locus segregated away in these plants and the targeted sites are thus stable (**Supplementary Figure S6**). However, the WGS analyses detected the presence of the vector backbone (**Supplementary Figure S7**) sequences at a specific locus on chromosome 8 in all plants of these plants (**Table 1**). Therefore, the generational nomenclature for these plants remained in the T_x_M_x_ form. Resequencing analysis indicated that the entire vector backbone, roughly 10 kb of DNA, was included in this insertion. When examined in greater detail, it was determined that the vector backbone insertion was present in the heterozygous state in the T_0_M_0_ plant WPT677-3 (**Table 1**). It appears that the backbone insertion segregated in the subsequent generation, exhibiting both heterozygous (4 plants) and homozygous (1 plant) progeny among the five sequenced T_1_M_1_ plants (**Table 1**). As the target gene (Glyma.08G084300) and the backbone insertion are located 11.4 Mb from one another on chromosome 8, it is possible that some of the edits are genetically linked to this backbone insertion. However, this is unlikely to be true for all edits, as the respective edits and the backbone insertion may be located on homologous chromosomes in some cases. The one plant identified as homozygous for the backbone insertion (WPT677-3-48) exhibited a heterozygous edited allele (**Table 1**), indicating that this particular mutation was not perfectly linked to the backbone. Presumably, some WPT677-3 descendants segregated out the backbone insertion while maintaining edited alleles of Glyma.08G084300, however no such lines were selected for WGS in this study.

*GmKASI* has a paralog copy (Glyma.05g129600) in the soybean genome. RNA expression of both *GmKASI* and its paralog copy have similar transcript expression profiles across all tissues (**Supplementary Figure S8A**; data extracted from Severin et al., 2010). This suggests that the paralog copy may have functional redundancy with *GmKASI*. We sought to investigate if our gRNAs exhibited any off-target mutagenesis to this paralog copy. The two gRNAs for target site-1 and target site-2 in *GmKASI* have three and two mismatches, respectively, when compared to the paralog copy (**Supplementary Figure S8B**). We sequenced PCR products of the paralog copy for both gRNA sites in plants WPT677-3-22, WPT677-35, WPT677-3-43, WPT677-3-44, WPT677-3-47, and WPT677-3-48. No evidence of new mutations were detected at either site in any of the assessed plants (**Supplementary Figure S8C**).

### Both Knockout and In-Frame Edited Alleles of GmKASI Altered Seed Morphology and Seed Composition Traits in Soybean

**Figure 3:**
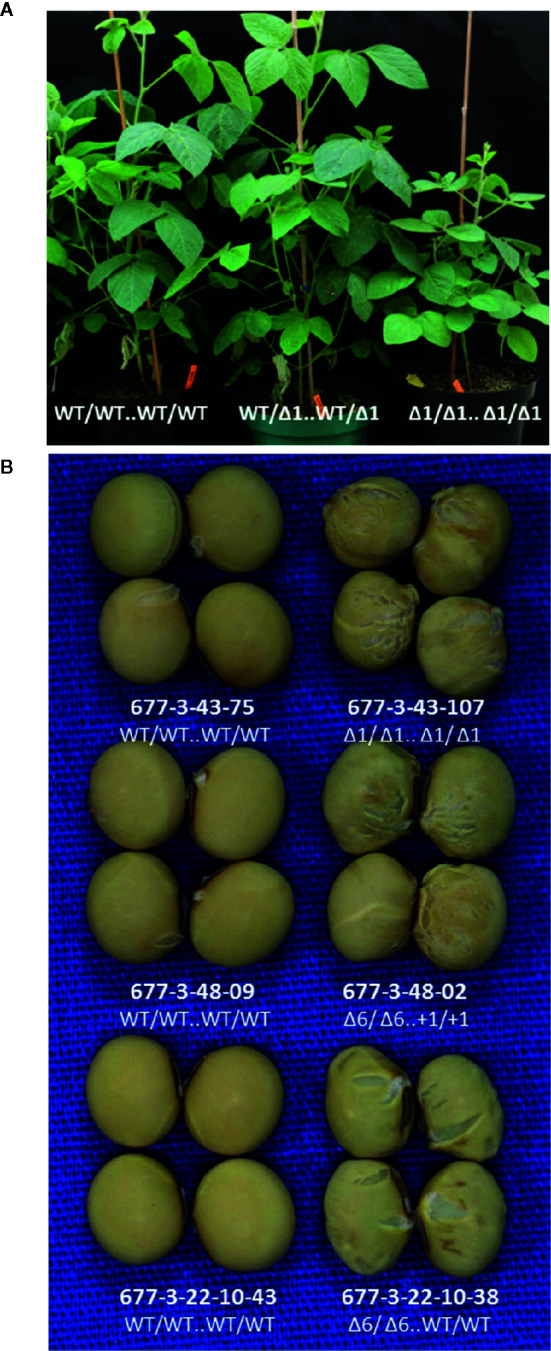
Both knockout and in-frame edited alleles of *GmKASI* displayed seed phenotypes**(A)** Representative plants of segregating T_2_M_2_ progeny from the heterozygous parental 677-3-43 plant. Plants carrying the *GmKASI* edited allele in the homozygous state displayed slow growth and semi-dwarf phenotypes. The genotype of each T_2_M_2_ plant is depicted as “*edit at target site 1 .. edit at target site 2*.” **(B)** Sample of seeds from homozygous wild-type and homozygous mutant plants segregating from three different heterozygous plants; 677-3-43, 677-3-48, and 677-3-22-10. Wild-type segregants are shown on the left, while homozygous mutant segregants are shown on the right. Plants 677-3-43 and 677-3-48 carried knockout edited alleles, while plant 677-3-22-10 carried an in-frame edited allele. Homozygous mutant seeds from homozygous knockout and in-frame alleles showed similar wrinkled and cracked seed morphologies.

We evaluated lines with edited alleles of *GmKASI*, wild-type segregants, and nontransformed cv. Bert for morphological and seed composition phenotypes based on two growouts in the greenhouse. Plants carrying homozygous mutant alleles displayed a range of slow growth compared to homozygous wild type siblings at the seedling stage (**Supplementary Figure S9**) and maintained slower growth rates at later stages (**Figure 3A**). Homozygous knock out and in-frame mutant plants both showed wrinkled and shriveled seed phenotypes (**Figure 3B**; **Supplementary Figure S10**) consistent with the fast neutron mutants described by Dobbels et al. (2017). Similar seed phenotypes were also confirmed in T_2_M_2_ seeds harvested from descendants of a separate CRISPR lineage derived from the T_0_M_0_ plant WPT677-6 (data not shown). The progeny from two WPT677-3 mutant lineages (WPT677-3-43-69 and WPT677-3-48-06) appeared to have many seeds with relatively normal shapes compared to the other mutants. However, both WPT677-3-43-69 and WPT677-3-48-06 carried heterozygous wild-type alleles at site 1 and site 2, therefore many of their seed progeny would be expected to carry a combination of wild type alleles at these two sites, rendering such seeds developmentally normal.

**Figure 4:**
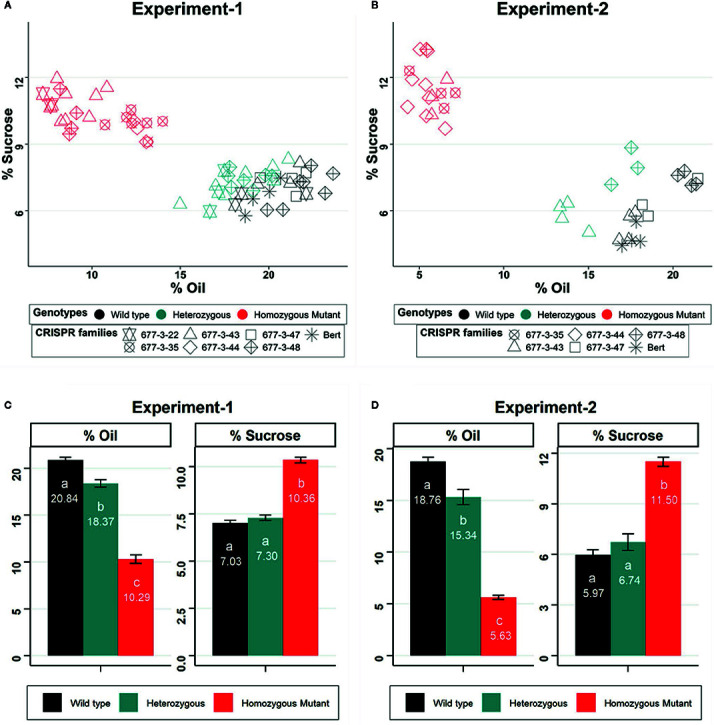
*GmKASI *regulates sucrose and oil content in soybean seeds **(A**, **B)** Scatter biplot of percent total oil and sucrose content for multiple CRISPR families (denoted by different symbols) based on NIR prediction. Data are shown from two independent greenhouse experiments. The genotypes are shown by different colors and refer to the parent plant that produced the seed. The parents were either homozygous for a mutation, heterozygous, or homozygous wild type. Three to 17 biological replicates were measured for each family. **(C**, **D)** Barplot of mean oil and sucrose content for each of the seed progeny from wild-type, heterozygous, and mutant genotypes in the two experiments. Homozygous mutants from all families in both experiments showed an increase in sucrose and a decrease in total oil content. Oil and sucrose measurements are based on the percent of seed, on a dry matter basis. One-way ANOVA and post-hoc Tukey-HSD (*alpha* = 0.01) tests were conducted. Genotypes with significantly different means are indicated by different letters (a, b, c). Error bars represent the mean ± standard error for each genotype class.

We analyzed seed composition traits from lineages of the WPT677-3 edited families of *GmKASI*, including wild-type segregants, and the nontransformed cv. Bert as controls, in two independent NIR experiments. To confirm the genotypes of individual plants, we genotyped each individual in each replicate. Detailed information about the genotype of individual plants from each CRISPR/Cas9 family are provided in **Supplementary Tables S1** and **S2**. In the first experiment, we evaluated six CRISPR/Cas9 families carrying one wild type, one in-frame, and six knock out alleles of *GmKASI*. WPT677-3-35 and WPT677-3-44 are biallelic mutants that produced families with all mutant plants. WPT677-3-43 (knock out), WPT677-3-48 (knock out) and WPT677-3-22-10 (in-frame) were heterozygous plants that produced families consisting of segregating wild type, heterozygous, and homozygous mutant individuals. All the plants carrying homozygous mutant alleles displayed an increase in seed sucrose content and a decrease in total seed oil content (**Figure 4A**). The individuals with homozygous in-frame alleles showed nearly identical seed phenotypes as the homozygous knockout individuals. On average, the homozygous mutant plants from all the families exhibited significantly higher sucrose content (10.36% on dry matter basis *vs*. a wild type value of 7.03%) and lower oil content (10.29% on dry matter basis *vs.* a wild type value of 20.84%) compared to homozygous wild type individuals in the first replicate (**Figure 4C**). The second experiment of this study showed similar results, although the extent of alteration in sucrose and oil contents were greater than in the first experiment (**Figures 4B, D**). Homozygous mutant plants showed an increase of sucrose content from 5.97% to 11.50% and a reduction of oil content from 18.76% to 5.63% as compared to wild type. Among other seed composition traits, homozygous mutant plants also showed a significant increase in linolenic fatty acid (*experiment 1*: from 8.55% to 15.48% of total fat; *experiment 2:* from 7.51% to 16.99% of total fat) and a decrease in linoleic fatty acid (*experiment 1*: from 49.42% to 30.57% of total fat; *experiment 2:* from 52.56% to 44.56% of total fat) compared to wild type (**Supplementary Figures S11** and **S12**).

### Non-Mendelian Inheritance of *GmKASI* Edited Alleles in Soybean

A low recovery of homozygous mutant segregants was observed in all three independent heterozygous lineages (progeny from WPT677-3-43, WPT677-3-48, and WPT677-3-22-10). In all cases, segregating progeny showed non-Mendelian inheritance with lower than expected transmission of the mutant alleles of *GmKASI* (**Table 2**). The segregation ratio significantly deviated from the expected 1:2:1 ratio in all three families (chi^2^ p value <0.001), with a particularly low recovery of homozygous mutant individuals.

**Table 2:** Non-Mendelian inheritance of *GmkasI* (Glyma.08G084300) mutant alleles.

Plant-ID	Genotype	Planted	Germination	WT	Het	Mut	Chi^2^ value	Chi^2^ p-value
Bert	WT control	29	100.00%	29	0	0	NA	NA
677-3-22-10	Heterozygous	69	89.86%	27	32	3	18.645	0.00009
677-3-43	Heterozygous	162	96.91%	53	86	18	17.038	0.0002
677-3-48	Heterozygous	89	88.76%	28	45	6	13.785	0.00102

“WT,” “Het,” and “Mut” are respectively the wild-type, heterozygous, and homozygous mutant segregants derived from a heterozygous parental plant.

## Discussion

This study set out to resolve three standing questions that resulted from previous work on *GmKasI* (Dobbels et al., 2017). First, would CRISPR mutagenesis validate the seed composition function of the *GmKASI* gene (Glyma.08G084300), and would the phenotype be different from the fast neutron line? This question was clearly addressed in the current study, as the seed composition phenotypes observed among a range of different CRISPR mutant lines were very similar to those observed in the previously published fast neutron translocation mutant of GmKasI (Dobbels et al., 2017). Compared to wild type seeds, the homozygous mutant seeds exhibited a wrinkled surface phenotype, an increase in percentage sucrose, a decrease in oil, and a redistribution of some fatty acid levels. Therefore, the CRISPR lines validated the function of *GmKasI* in determining the seed morphology and seed composition phenotypes. It is important to note that the phenotypic analysis of the WPT677-3 family may have been complicated by the presence of a vector backbone insertion in many of these plants. However, there is ample evidence indicating that the vector backbone insertion did not influence the mutant phenotypes observed in the WPT677-3 mutant descendants. First, plant WPT677-3-47, which carried wild-type alleles for *GmKASI* and was heterozygous for the backbone insertion (**Table 1**), only produced wild type (not wrinkled) seeds. Second, T_2_M_2_ seeds harvested from descendants of the WPT677-6 family showed numerous wrinkled individuals, while presumably not carrying the backbone insertion, as they were derived from an independent T_0_ event. These findings further confirm that the mutant phenotypes in this study are caused by the mutations at *GmKasI*.

Progeny from heterozygous mutant individuals typically displayed an intermediate mean phenotype for the seed composition traits (**Figure 4**). However, this is likely an outcome of the need to pool multiple individuals in each NIR run. We hypothesize that the progeny from the heterozygous lines consists of a combination of homozygous wild-type, heterozygous, and homozygous mutant individuals. Each individual seed likely displayed the composition traits of either the wild type group (presumably this includes both homozygous wild type and heterozygous individuals) or the extreme mutant phenotype (for homozygous mutant individuals). However, the pooled combination shows an intermediate phenotype. The traits tend to be more similar to the wild type composition presumably because the transmission of the homozygous mutant type is relatively low compared to the other groups (**Table 2**). It is also noteworthy that differences were observed in the severity of the seed composition traits between the first and second greenhouse experiments. While the experiments were grown using the same families and in the same greenhouse, they were grown at different times. These differences between experiments suggest that seed composition traits are influenced by microenvironment differences, even in greenhouse conditions.

The second question of interest addressed the possibility that an in-frame mutation of *GmKasI* may provide a less extreme (i.e., intermediate) seed composition phenotype compared to the knockout mutations. This idea was previously demonstrated in a series of soybean trichome mutants—the in-frame alleles of a *CPR5* gene ortholog demonstrated an intermediate phenotype compared to the wild type and knockout mutants (Campbell et al., 2019). This “weak allele” concept is a promising avenue for using gene editing as a means to develop agriculturally useful phenotypes in cases where full knockout alleles exhibit phenotypes that are too extreme and/or cause secondary undesirable phenotypes. However, the data from our in-frame mutant lines did not support this outcome in the present study. The mutant individuals carrying the homozygous in-frame allele also exhibited very strong alterations in seed sucrose and oil content, comparable to the knockout lines. Thus, an intermediate (i.e., less severe) phenotype was not observed in the in-frame mutant. However, it is notable that the in-frame mutation was in a highly evolutionarily conserved region (**Supplementary Figure S1**). It may have been expected that perturbations to amino acids in such a conserved region may have strong phenotypic consequences. Perhaps in-frame mutations in a less conserved domain of *GmKasI* would yield intermediate phenotypes.

The third question of interest addressed the low transmission of soybean *kasI* mutant alleles. While reduced transmission of the *kasI* mutant was observed in the fast neutron line, this particular mutation was linked to a translocation event that may have also disrupted meiosis. However, the progeny of the CRISPR lines in the current study heterozygous for the mutations also exhibited low transmission of the mutated alleles, while showing no indications of chromosomal abnormalities. Thus, we conclude that the low transmission of *GmkasI* mutations is a consequence of the mutations *per se*. While we do not know the mechanism for the reduced transmission, it is possible that post-fertilization defects during embryogenesis or seed development may lead to lethality in some of the homozygous mutant individuals.

Taken together, these results from this study confirm that *GmKASI* is an important gene for regulating the sucrose to oil biosynthesis pathway in soybean. This is a function that has also been demonstrated in Arabidopsis (Wu and Xue, 2010). This observation has now been made across three different mutant classes of soybean, including a translocation line, CRISPR knockout alleles, and a CRISPR in-frame allele.

## Data Availability Statement

The datasets presented in this study can be found in online repositories. The names of the repository/repositories and accession number(s) can be found below: https://www.ncbi.nlm.nih.gov/, PRJNA640373.

## Author Contributions

All authors contributed to the article and approved the submitted version. KV, GM, and RS conceived and designed the experiments. KV conducted the CRISPR experiments and analyzed the data. MS contributed to genotyping and greenhouse experiments. AS contributed to Sanger sequencing and heteroduplex assays. YX generated transformed plants. RM conducted whole genome sequencing bioinformatics. KV and RS drafted the manuscript.

## Funding

This work was supported in part by the Minnesota Soybean Research and Promotion Council (projects #150-4140-18-02 and #10-15-47-19-174-7527), and by an endowed chair of molecular genetics to crop improvement.

## Conflict of Interest

The authors declare that the research was conducted in the absence of any commercial or financial relationships that could be construed as a potential conflict of interest.
