# Supplementary material for: Similar Seed Composition Phenotypes Are Observed From CRISPR-Generated In-Frame and Knockout Alleles of a Soybean KASI Ortholog
Source: Front Plant Sci. 2020 Jul 8;11:1005. doi: 10.3389/fpls.2020.01005 (PMC7381328; doi:10.3389/fpls.2020.01005)
Supplement: Supplementary file 4 [file Presentation_1.pptx]

## Slide 1
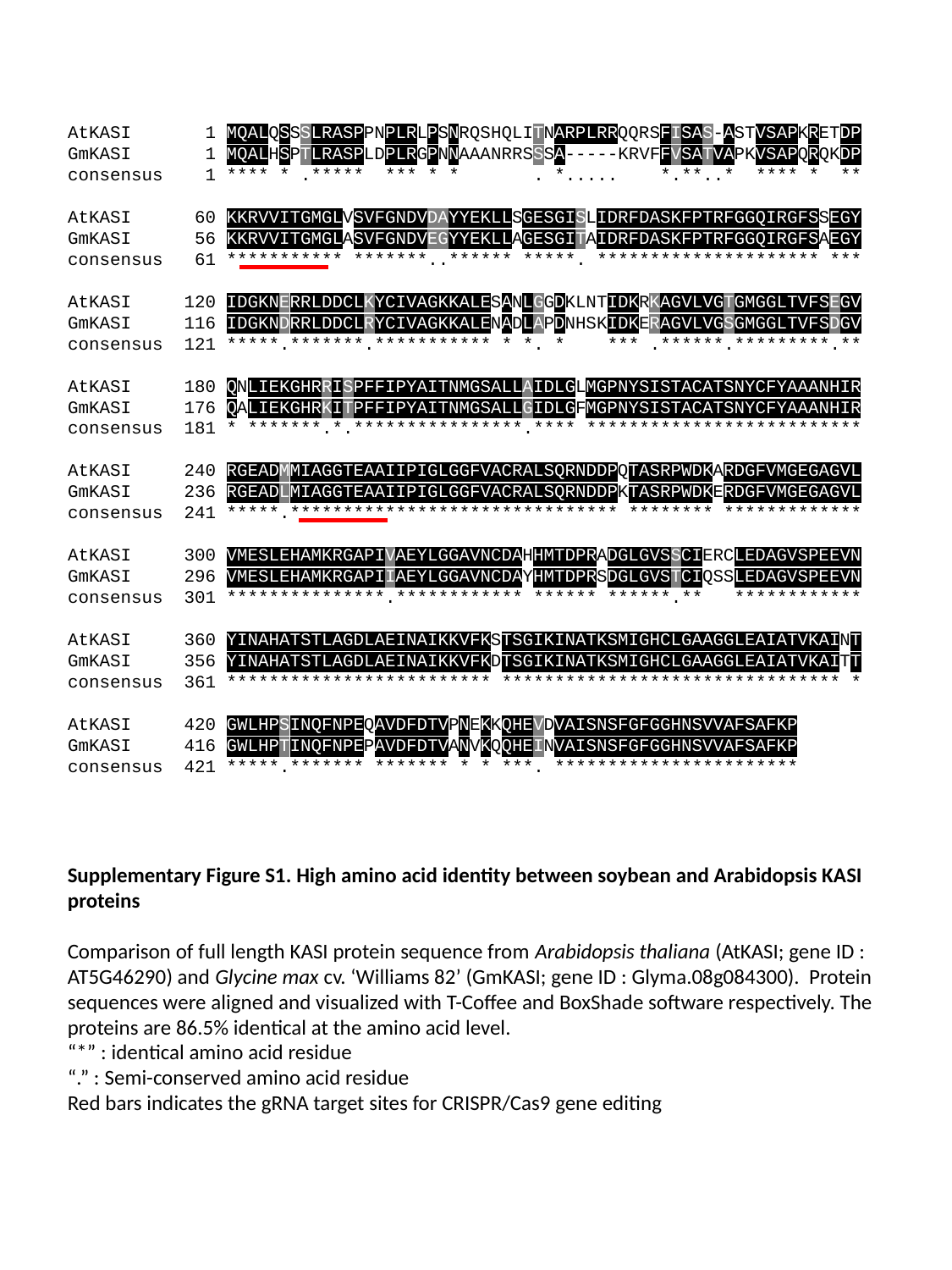

AtKASI 1 MQALQSSSLRASPPNPLRLPSNRQSHQLITNARPLRRQQRSFISAS-ASTVSAPKRETDP
GmKASI 1 MQALHSPTLRASPLDPLRGPNNAAANRRSSSA-----KRVFFVSATVAPKVSAPQRQKDP
consensus 1 **** * .***** *** * * . *..... *.**..* **** * **
AtKASI 60 KKRVVITGMGLVSVFGNDVDAYYEKLLSGESGISLIDRFDASKFPTRFGGQIRGFSSEGY
GmKASI 56 KKRVVITGMGLASVFGNDVEGYYEKLLAGESGITAIDRFDASKFPTRFGGQIRGFSAEGY
consensus 61 *********** *******..****** *****. ********************* ***
AtKASI 120 IDGKNERRLDDCLKYCIVAGKKALESANLGGDKLNTIDKRKAGVLVGTGMGGLTVFSEGV
GmKASI 116 IDGKNDRRLDDCLRYCIVAGKKALENADLAPDNHSKIDKERAGVLVGSGMGGLTVFSDGV
consensus 121 *****.*******.*********** * *. * *** .******.*********.**
AtKASI 180 QNLIEKGHRRISPFFIPYAITNMGSALLAIDLGLMGPNYSISTACATSNYCFYAAANHIR
GmKASI 176 QALIEKGHRKITPFFIPYAITNMGSALLGIDLGFMGPNYSISTACATSNYCFYAAANHIR
consensus 181 * *******.*.****************.**** **************************
AtKASI 240 RGEADMMIAGGTEAAIIPIGLGGFVACRALSQRNDDPQTASRPWDKARDGFVMGEGAGVL
GmKASI 236 RGEADLMIAGGTEAAIIPIGLGGFVACRALSQRNDDPKTASRPWDKERDGFVMGEGAGVL
consensus 241 *****.******************************* ******** *************
AtKASI 300 VMESLEHAMKRGAPIVAEYLGGAVNCDAHHMTDPRADGLGVSSCIERCLEDAGVSPEEVN
GmKASI 296 VMESLEHAMKRGAPIIAEYLGGAVNCDAYHMTDPRSDGLGVSTCIQSSLEDAGVSPEEVN
consensus 301 ***************.************ ****** ******.** ************
AtKASI 360 YINAHATSTLAGDLAEINAIKKVFKSTSGIKINATKSMIGHCLGAAGGLEAIATVKAINT
GmKASI 356 YINAHATSTLAGDLAEINAIKKVFKDTSGIKINATKSMIGHCLGAAGGLEAIATVKAITT
consensus 361 ************************* ******************************** *
AtKASI 420 GWLHPSINQFNPEQAVDFDTVPNEKKQHEVDVAISNSFGFGGHNSVVAFSAFKP
GmKASI 416 GWLHPTINQFNPEPAVDFDTVANVKQQHEINVAISNSFGFGGHNSVVAFSAFKP
consensus 421 *****.******* ******* * * ***. ***********************
Supplementary Figure S1. High amino acid identity between soybean and Arabidopsis KASI proteins
Comparison of full length KASI protein sequence from Arabidopsis thaliana (AtKASI; gene ID : AT5G46290) and Glycine max cv. ‘Williams 82’ (GmKASI; gene ID : Glyma.08g084300). Protein sequences were aligned and visualized with T-Coffee and BoxShade software respectively. The proteins are 86.5% identical at the amino acid level.
“*” : identical amino acid residue
“.” : Semi-conserved amino acid residue
Red bars indicates the gRNA target sites for CRISPR/Cas9 gene editing

## Slide 2
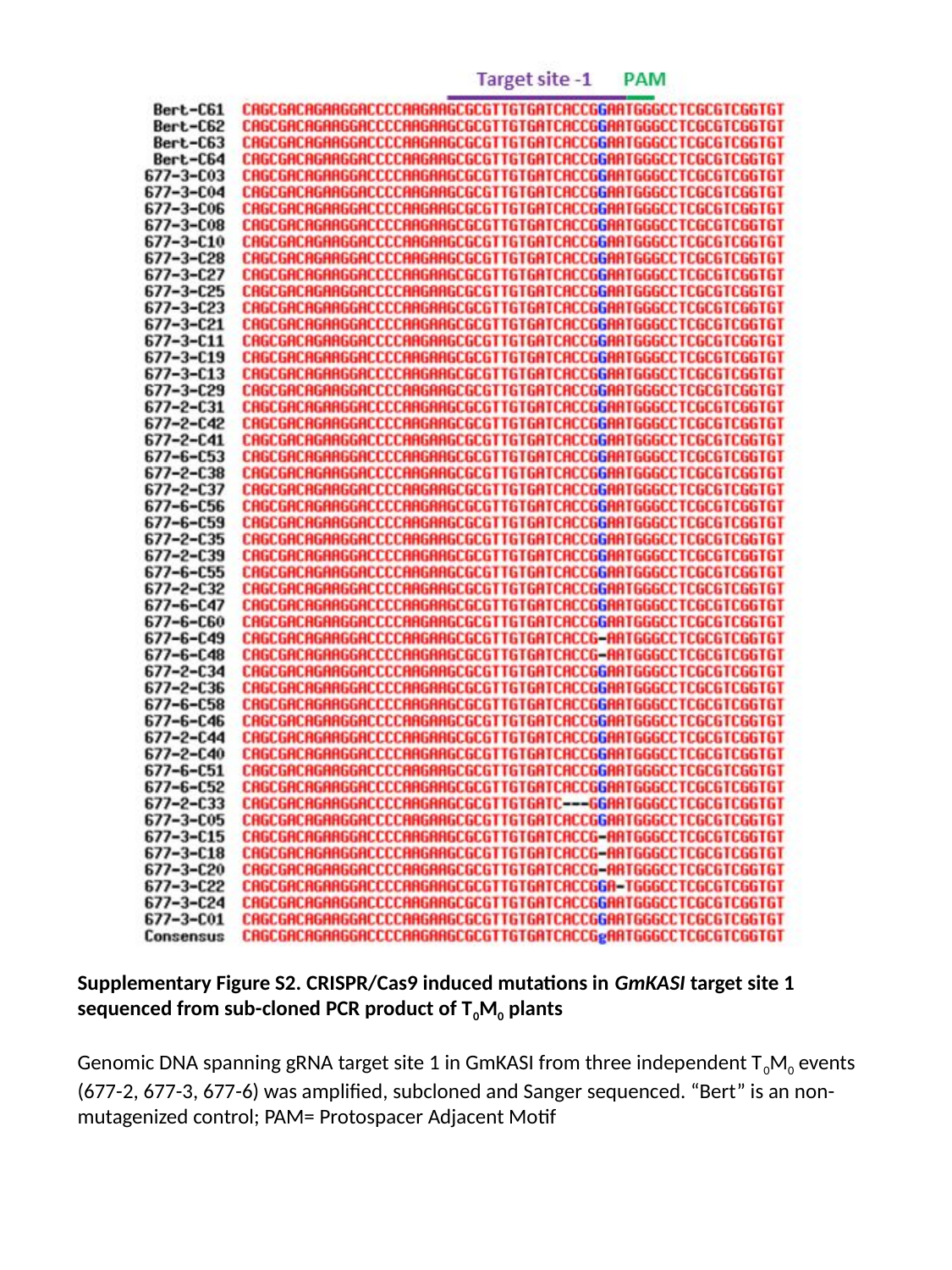

Supplementary Figure S2. CRISPR/Cas9 induced mutations in GmKASI target site 1 sequenced from sub-cloned PCR product of T0M0 plants
Genomic DNA spanning gRNA target site 1 in GmKASI from three independent T0M0 events (677-2, 677-3, 677-6) was amplified, subcloned and Sanger sequenced. “Bert” is an non-mutagenized control; PAM= Protospacer Adjacent Motif

## Slide 3
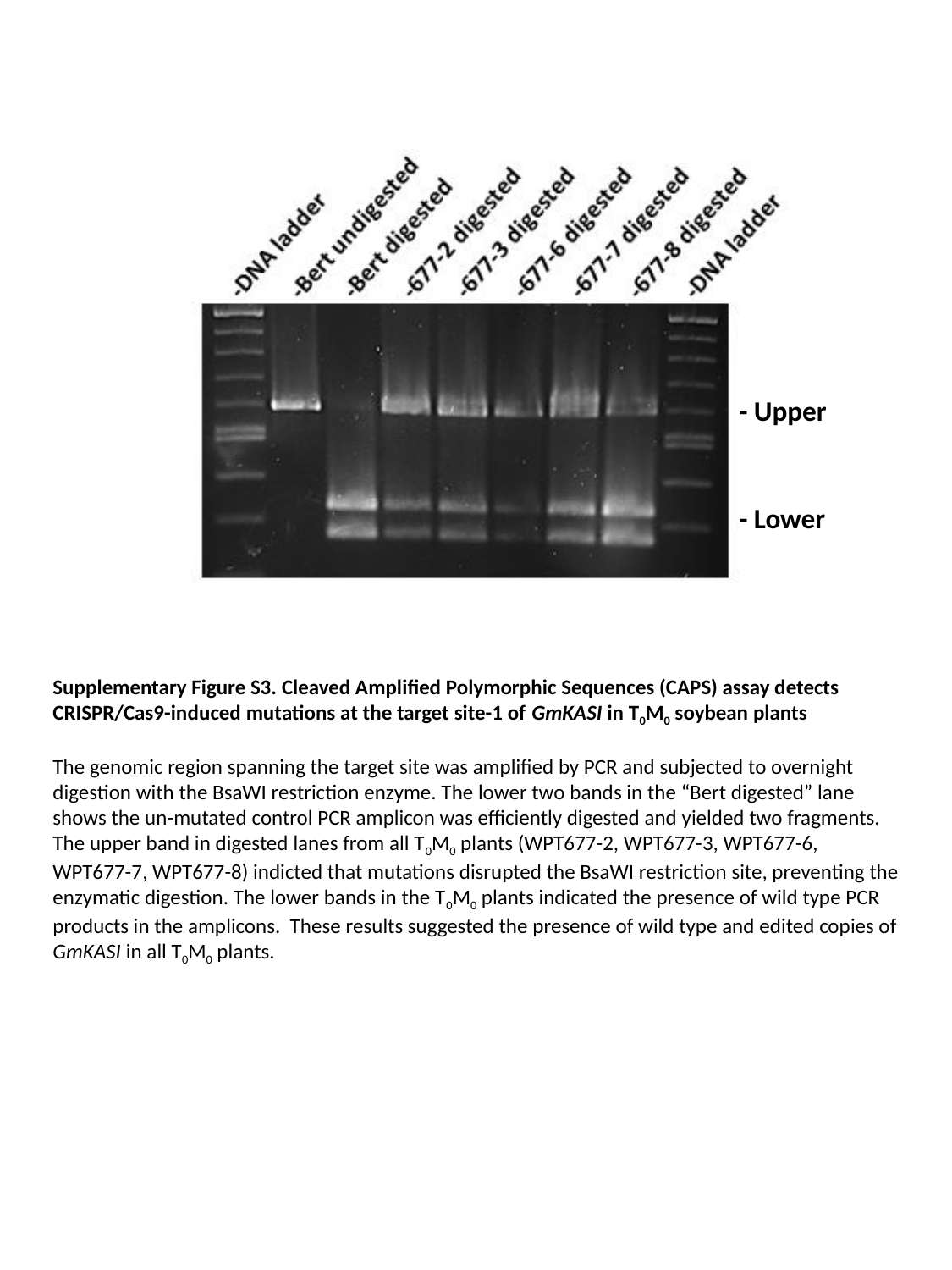

- Upper
- Lower
Supplementary Figure S3. Cleaved Amplified Polymorphic Sequences (CAPS) assay detects CRISPR/Cas9-induced mutations at the target site-1 of GmKASI in T0M0 soybean plants
The genomic region spanning the target site was amplified by PCR and subjected to overnight digestion with the BsaWI restriction enzyme. The lower two bands in the “Bert digested” lane shows the un-mutated control PCR amplicon was efficiently digested and yielded two fragments. The upper band in digested lanes from all T0M0 plants (WPT677-2, WPT677-3, WPT677-6, WPT677-7, WPT677-8) indicted that mutations disrupted the BsaWI restriction site, preventing the enzymatic digestion. The lower bands in the T0M0 plants indicated the presence of wild type PCR products in the amplicons. These results suggested the presence of wild type and edited copies of GmKASI in all T0M0 plants.

## Slide 4
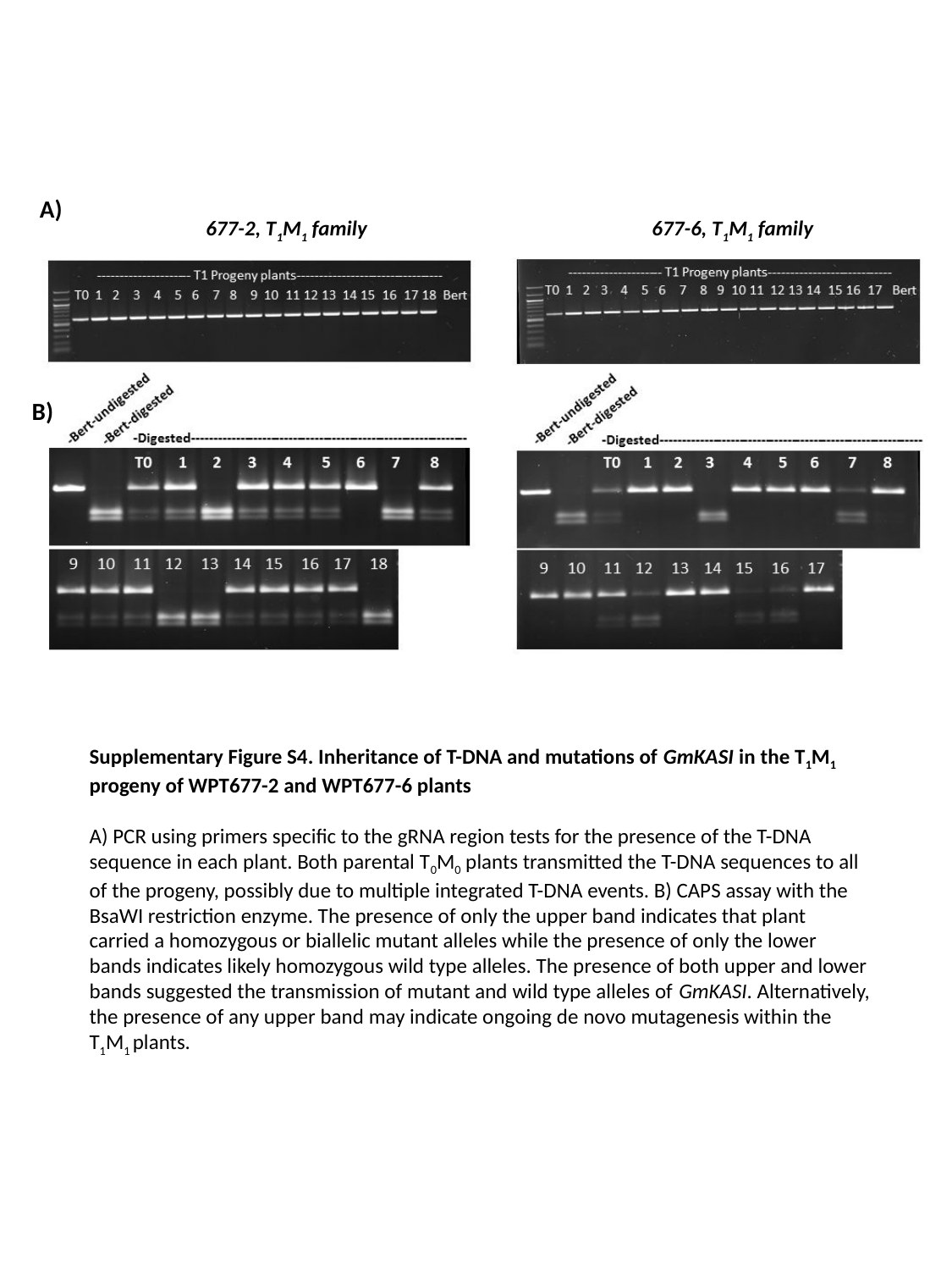

A)
677-2, T1M1 family
677-6, T1M1 family
B)
Supplementary Figure S4. Inheritance of T-DNA and mutations of GmKASI in the T1M1 progeny of WPT677-2 and WPT677-6 plants
A) PCR using primers specific to the gRNA region tests for the presence of the T-DNA sequence in each plant. Both parental T0M0 plants transmitted the T-DNA sequences to all of the progeny, possibly due to multiple integrated T-DNA events. B) CAPS assay with the BsaWI restriction enzyme. The presence of only the upper band indicates that plant carried a homozygous or biallelic mutant alleles while the presence of only the lower bands indicates likely homozygous wild type alleles. The presence of both upper and lower bands suggested the transmission of mutant and wild type alleles of GmKASI. Alternatively, the presence of any upper band may indicate ongoing de novo mutagenesis within the T1M1 plants.

## Slide 5
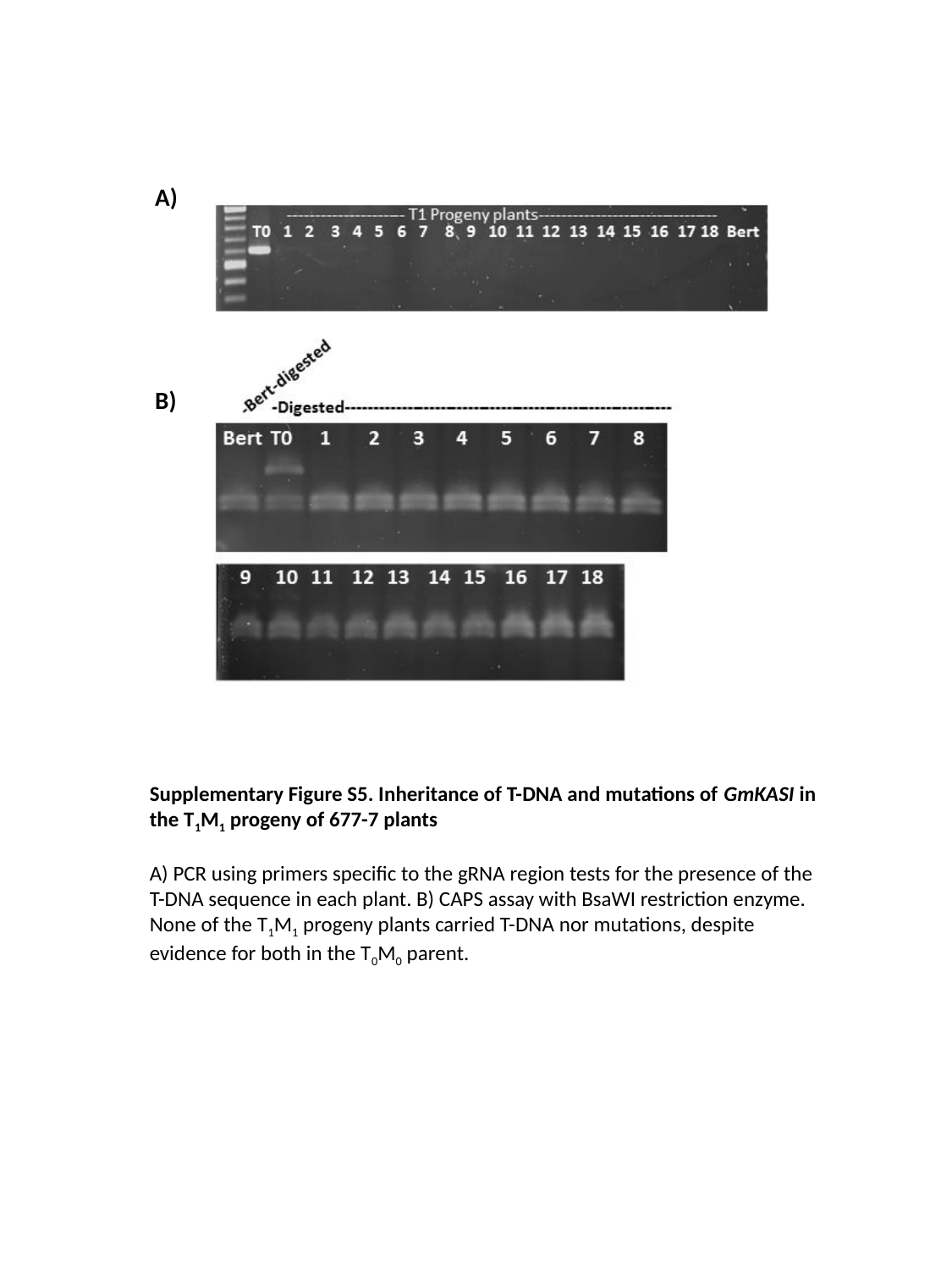

A)
B)
Supplementary Figure S5. Inheritance of T-DNA and mutations of GmKASI in the T1M1 progeny of 677-7 plants
A) PCR using primers specific to the gRNA region tests for the presence of the T-DNA sequence in each plant. B) CAPS assay with BsaWI restriction enzyme. None of the T1M1 progeny plants carried T-DNA nor mutations, despite evidence for both in the T0M0 parent.

## Slide 6
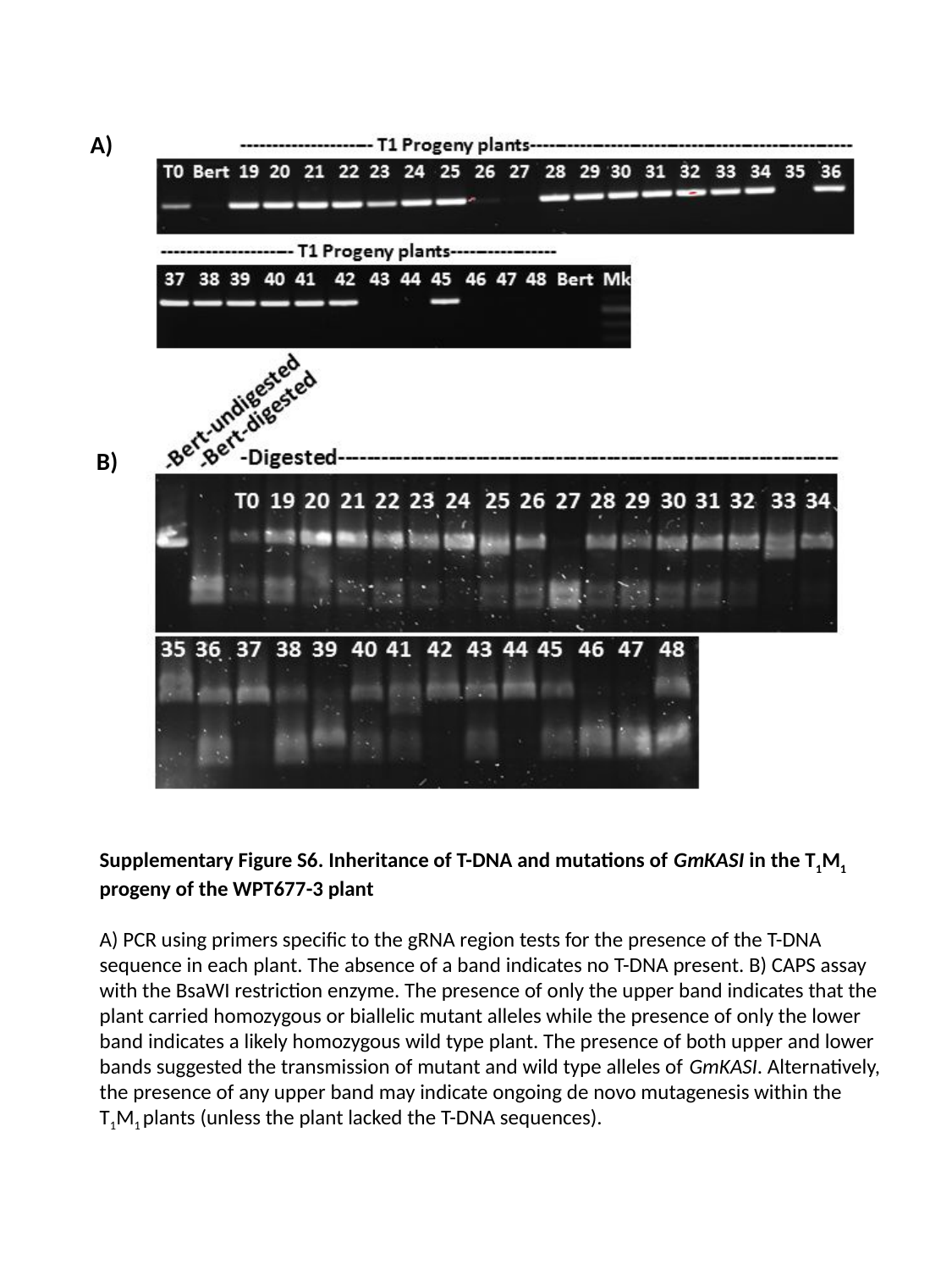

A)
B)
Supplementary Figure S6. Inheritance of T-DNA and mutations of GmKASI in the T1M1 progeny of the WPT677-3 plant
A) PCR using primers specific to the gRNA region tests for the presence of the T-DNA sequence in each plant. The absence of a band indicates no T-DNA present. B) CAPS assay with the BsaWI restriction enzyme. The presence of only the upper band indicates that the plant carried homozygous or biallelic mutant alleles while the presence of only the lower band indicates a likely homozygous wild type plant. The presence of both upper and lower bands suggested the transmission of mutant and wild type alleles of GmKASI. Alternatively, the presence of any upper band may indicate ongoing de novo mutagenesis within the T1M1 plants (unless the plant lacked the T-DNA sequences).

## Slide 7
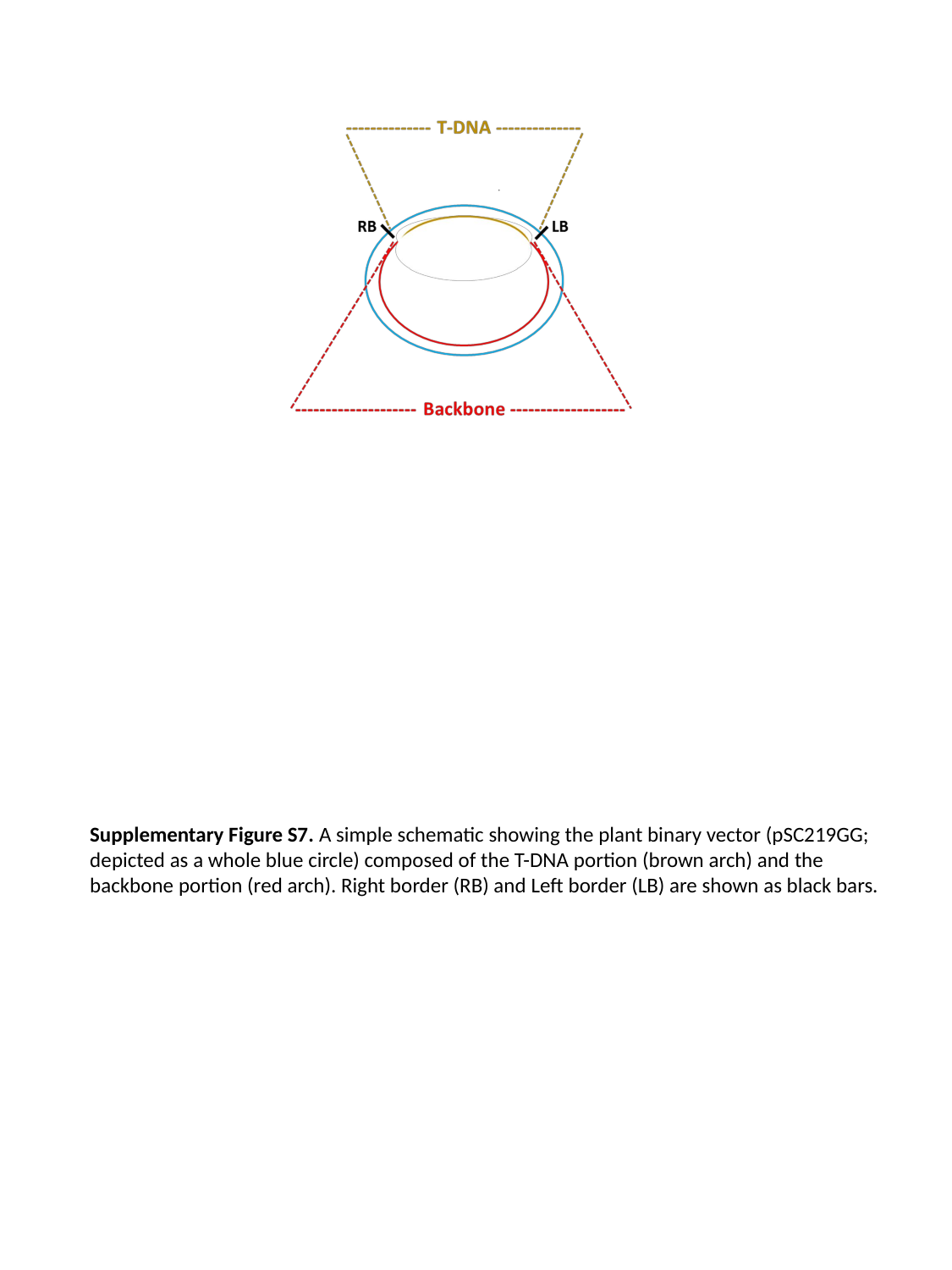

Supplementary Figure S7. A simple schematic showing the plant binary vector (pSC219GG; depicted as a whole blue circle) composed of the T-DNA portion (brown arch) and the backbone portion (red arch). Right border (RB) and Left border (LB) are shown as black bars.

## Slide 8
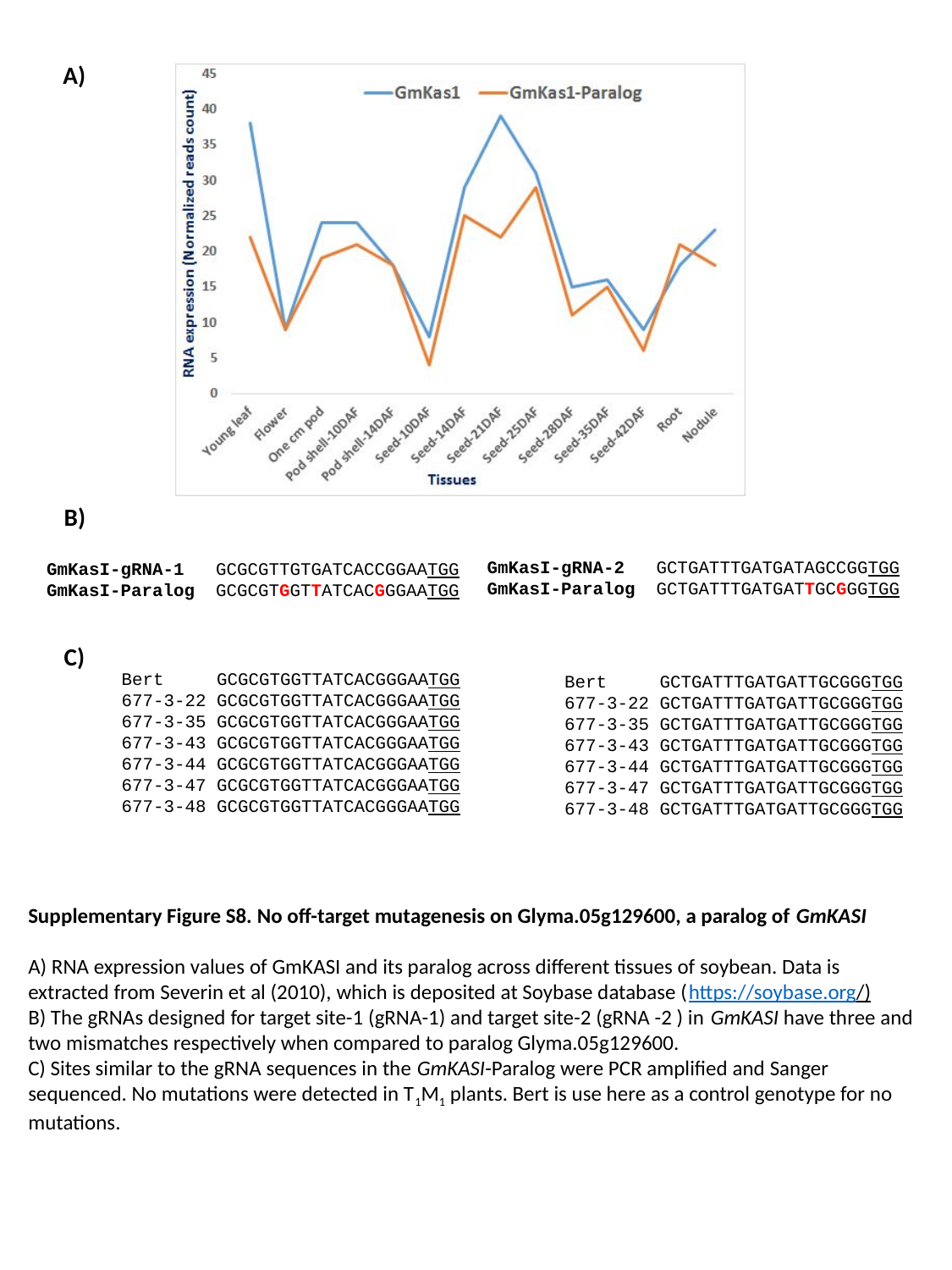

A)
B)
GmKasI-gRNA-2 GCTGATTTGATGATAGCCGGTGG
GmKasI-Paralog GCTGATTTGATGATTGCGGGTGG
GmKasI-gRNA-1 GCGCGTTGTGATCACCGGAATGG
GmKasI-Paralog GCGCGTGGTTATCACGGGAATGG
C)
Bert GCGCGTGGTTATCACGGGAATGG
677-3-22 GCGCGTGGTTATCACGGGAATGG
677-3-35 GCGCGTGGTTATCACGGGAATGG
677-3-43 GCGCGTGGTTATCACGGGAATGG
677-3-44 GCGCGTGGTTATCACGGGAATGG
677-3-47 GCGCGTGGTTATCACGGGAATGG
677-3-48 GCGCGTGGTTATCACGGGAATGG
Bert GCTGATTTGATGATTGCGGGTGG
677-3-22 GCTGATTTGATGATTGCGGGTGG
677-3-35 GCTGATTTGATGATTGCGGGTGG
677-3-43 GCTGATTTGATGATTGCGGGTGG
677-3-44 GCTGATTTGATGATTGCGGGTGG
677-3-47 GCTGATTTGATGATTGCGGGTGG
677-3-48 GCTGATTTGATGATTGCGGGTGG
Supplementary Figure S8. No off-target mutagenesis on Glyma.05g129600, a paralog of GmKASI
A) RNA expression values of GmKASI and its paralog across different tissues of soybean. Data is extracted from Severin et al (2010), which is deposited at Soybase database (https://soybase.org/)
B) The gRNAs designed for target site-1 (gRNA-1) and target site-2 (gRNA -2 ) in GmKASI have three and two mismatches respectively when compared to paralog Glyma.05g129600.
C) Sites similar to the gRNA sequences in the GmKASI-Paralog were PCR amplified and Sanger sequenced. No mutations were detected in T1M1 plants. Bert is use here as a control genotype for no mutations.

## Slide 9
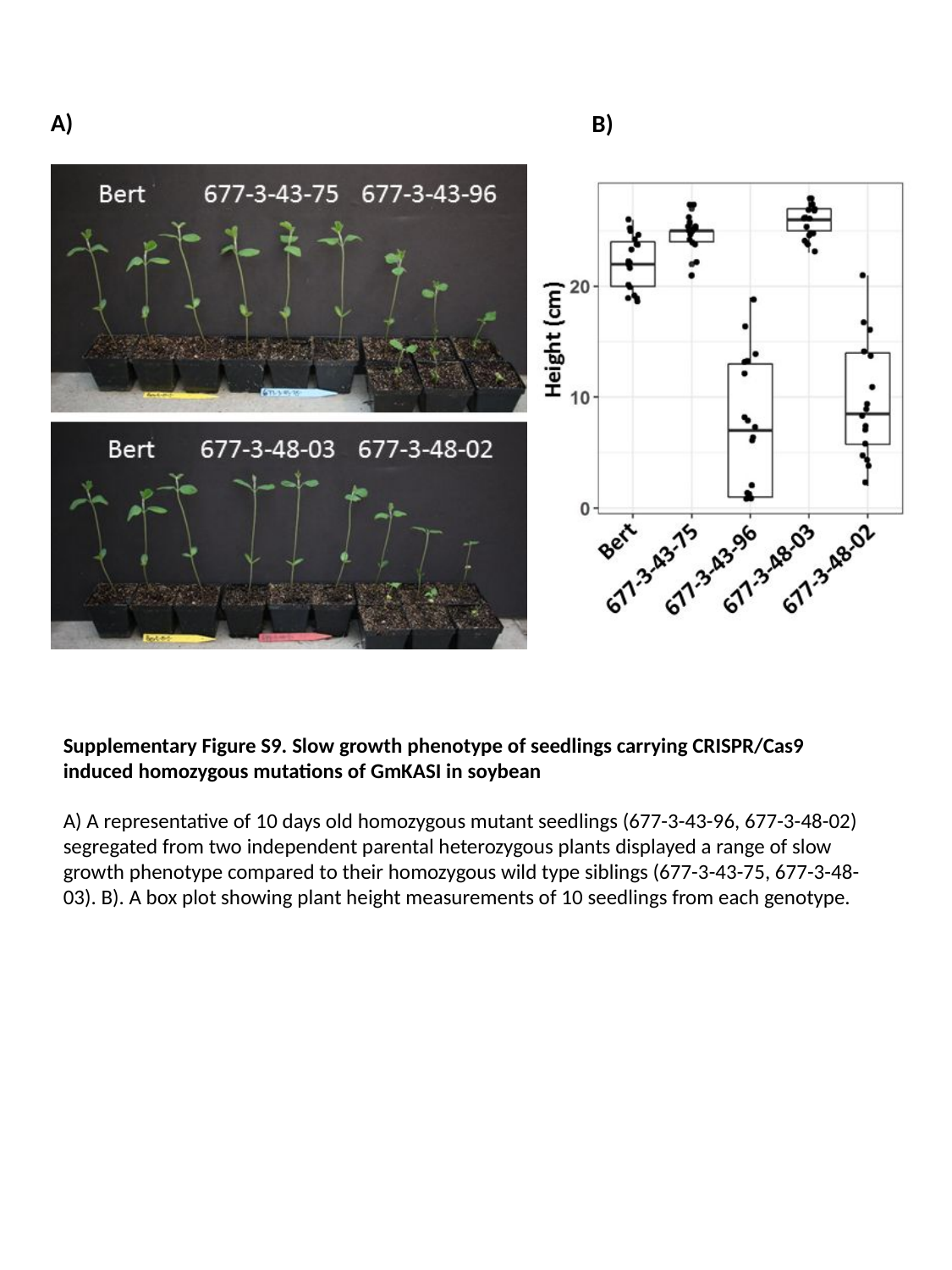

A)
B)
Bert 677-3-48-03 677-3-48-02
Supplementary Figure S9. Slow growth phenotype of seedlings carrying CRISPR/Cas9 induced homozygous mutations of GmKASI in soybean
A) A representative of 10 days old homozygous mutant seedlings (677-3-43-96, 677-3-48-02) segregated from two independent parental heterozygous plants displayed a range of slow growth phenotype compared to their homozygous wild type siblings (677-3-43-75, 677-3-48-03). B). A box plot showing plant height measurements of 10 seedlings from each genotype.
M92-220 M14-639-02 M14-639-150

## Slide 10
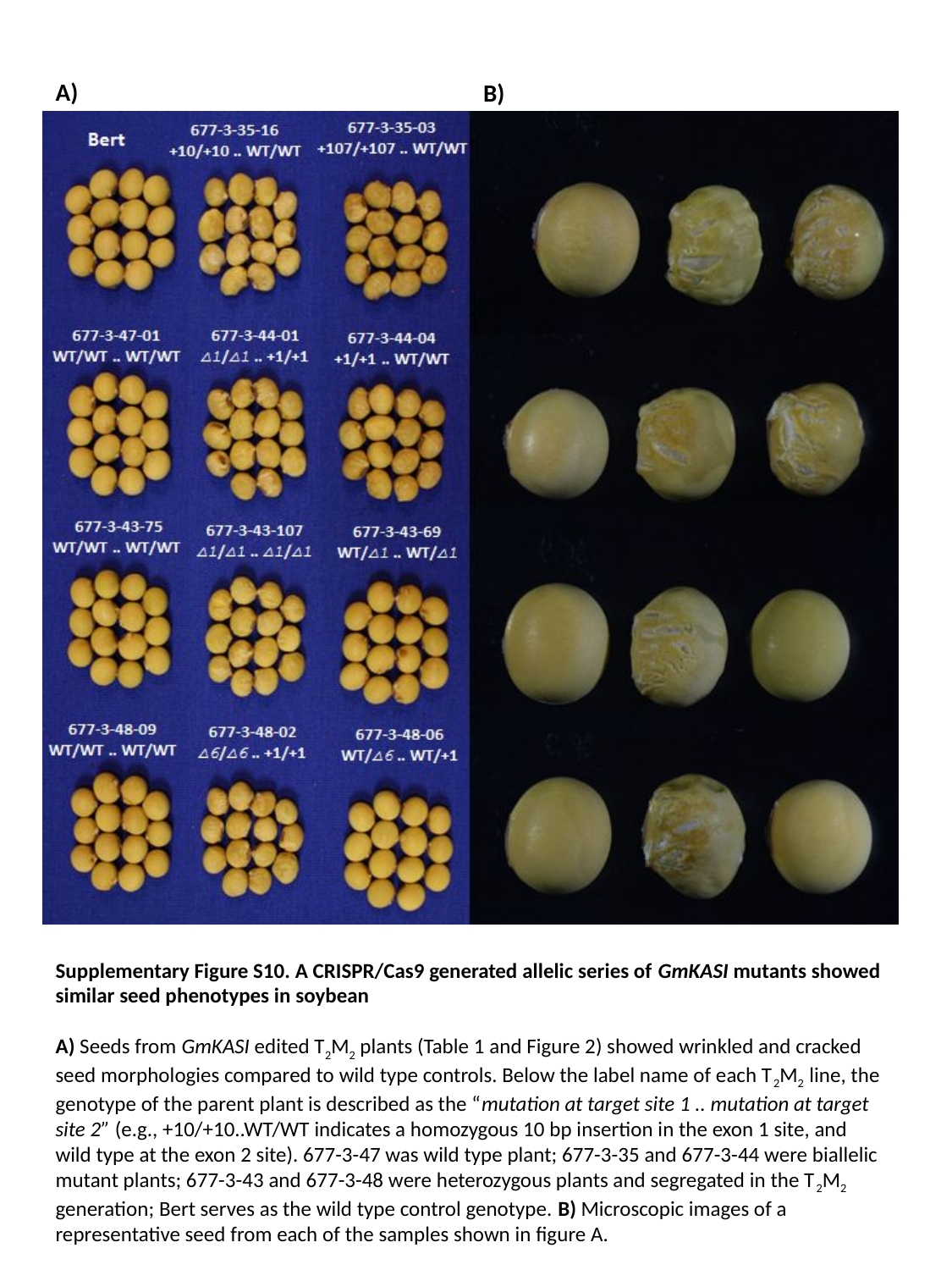

A)
B)
Supplementary Figure S10. A CRISPR/Cas9 generated allelic series of GmKASI mutants showed similar seed phenotypes in soybean
A) Seeds from GmKASI edited T2M2 plants (Table 1 and Figure 2) showed wrinkled and cracked seed morphologies compared to wild type controls. Below the label name of each T2M2 line, the genotype of the parent plant is described as the “mutation at target site 1 .. mutation at target site 2” (e.g., +10/+10..WT/WT indicates a homozygous 10 bp insertion in the exon 1 site, and wild type at the exon 2 site). 677-3-47 was wild type plant; 677-3-35 and 677-3-44 were biallelic mutant plants; 677-3-43 and 677-3-48 were heterozygous plants and segregated in the T2M2 generation; Bert serves as the wild type control genotype. B) Microscopic images of a representative seed from each of the samples shown in figure A.

## Slide 11
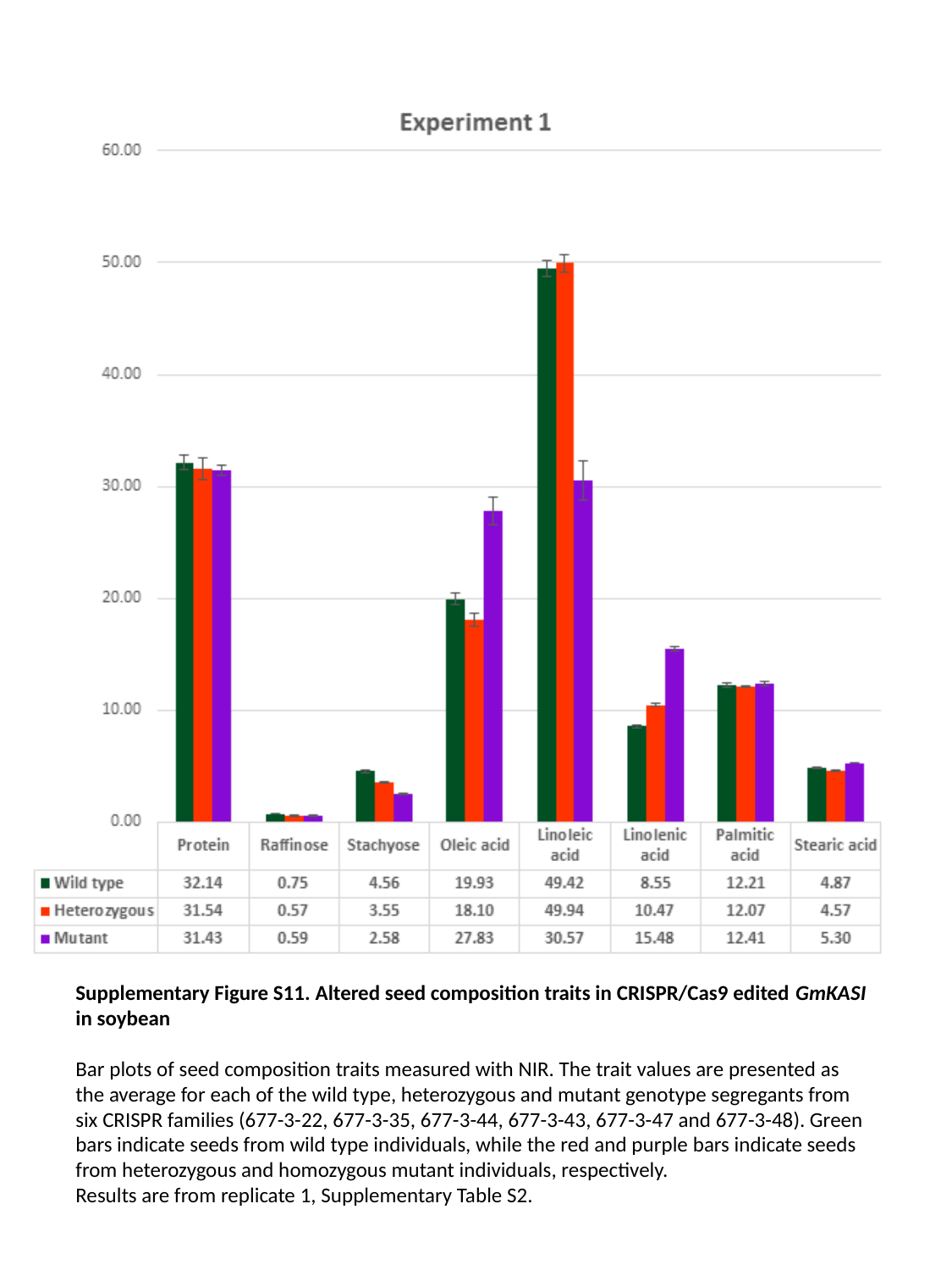

Supplementary Figure S11. Altered seed composition traits in CRISPR/Cas9 edited GmKASI in soybean
Bar plots of seed composition traits measured with NIR. The trait values are presented as the average for each of the wild type, heterozygous and mutant genotype segregants from six CRISPR families (677-3-22, 677-3-35, 677-3-44, 677-3-43, 677-3-47 and 677-3-48). Green bars indicate seeds from wild type individuals, while the red and purple bars indicate seeds from heterozygous and homozygous mutant individuals, respectively.
Results are from replicate 1, Supplementary Table S2.

## Slide 12
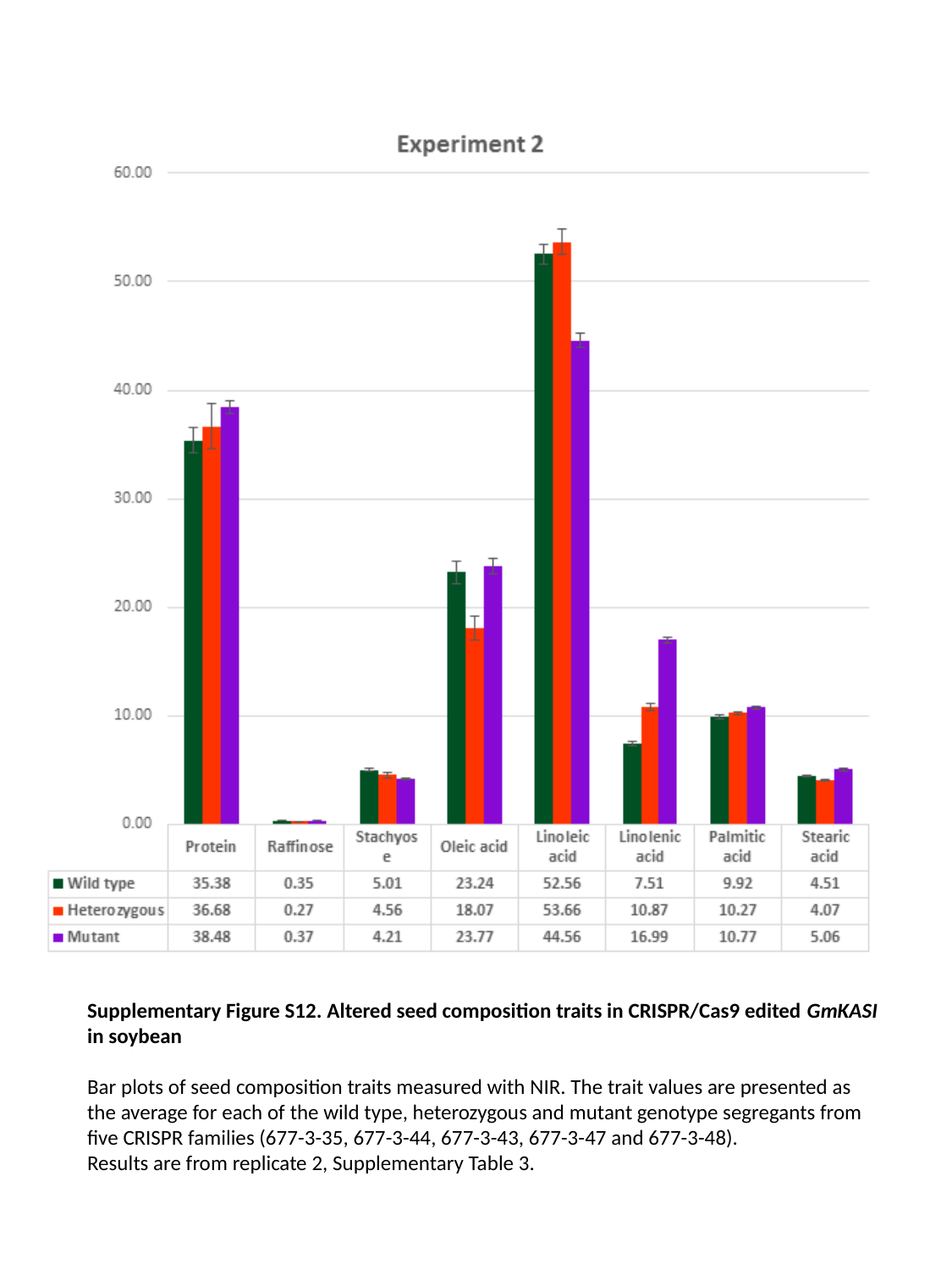

Supplementary Figure S12. Altered seed composition traits in CRISPR/Cas9 edited GmKASI in soybean
Bar plots of seed composition traits measured with NIR. The trait values are presented as the average for each of the wild type, heterozygous and mutant genotype segregants from five CRISPR families (677-3-35, 677-3-44, 677-3-43, 677-3-47 and 677-3-48).
Results are from replicate 2, Supplementary Table 3.
